# Defunctioning ileostomy reduces leakage rate in rectal cancer surgery - systematic review and meta-analysis

**DOI:** 10.18632/oncotarget.25015

**Published:** 2018-04-17

**Authors:** Magdalena Pisarska, Natalia Gajewska, Piotr Małczak, Michał Wysocki, Jan Witowski, Grzegorz Torbicz, Piotr Major, Magdalena Mizera, Marcin Dembiński, Marcin Migaczewski, Andrzej Budzyński, Michał Pędziwiatr

**Affiliations:** ^1^ 2^nd^ Department of General Surgery, Jagiellonian University Medical College, Kraków, Poland; ^2^ Centre for Research, Training and Innovation in Surgery (CERTAIN Surgery), Kraków, Poland

**Keywords:** defunctioning ileostomy, leakage, rectal cancer, meta-analysis

## Abstract

**Objectives:**

The role of a defunctioning ileostomy in every anterior rectal resection with total mesorectal excision (TME) is still controversial. In this study, we aimed to review the current literature to determine the impact of ileostomy creation on postoperative outcomes in patients undergoing anterior rectal resection with TME.

**Methods:**

MEDLINE, Embase and Cochrane Library were searched for eligible studies. We analyzed data up to October 2017. Eligible studies had to compare patients with vs. without a defunctioning ileostomy in rectal cancer surgery and comprise data on anastomotic leakage in both groups. The primary outcome was anastomotic leakage. Secondary outcomes included the complication rate, mortality, reoperation rate, length of hospital stay and 30-day readmission.

**Results:**

Initial search yielded 1,966 articles. Thorough evaluation resulted in 13 eligible articles which were analyzed. Leakage rate (RR = 0.43, 95% CI 0.28-0.67) and the number of reoperations (RR = 0.62, 95% CI 0.40-0.94) were significantly lower in the defunctioning stoma group. Morbidity was significantly higher in the stoma group (RR = 1.32, 95% CI 1.05–1.65). Analysis of mortality, length of hospital stay and readmission rate did not show any significant differences.

**Conclusion:**

A defunctioning ileostomy may decrease the anastomotic leakage rate, additionally significantly reducing the risk of reoperations but it may also increase the overall complication rate. The presence of the protective stoma has no effect on mortality, length of hospital stay and readmission rate.

## INTRODUCTION

The standard potentially curative treatment option for rectal cancer is surgery, which is often combined with preoperative radio or chemoradiotherapy [1]. In 1982 Heald introduced the total mesorectal excision (TME) that has become the standard technique to dissect in anatomical planes with the aim to obtain a complete removal of mesorectum with intact mesorectal fascia [2]. Despite improvements in surgical technique and development of modern equipment, including laparoscopic surgery, TME with primary anastomosis is still associated with a significant risk of symptomatic anastomotic leakage (AL), ranging between 3% and 24% [3–5]. The rate depends mainly on the tumor size and location, neoadjuvant irradiation and patient’s general status (male gender, malnutrition, steroid use, obesity and advanced age are all associated with increased risk) [6–8]. Patients with low rectal cancer are prone to higher rate of intraoperative adverse events and permanent colostomy which results in worse functional outcome compared with high rectal cancer [9, 10]. The prevention of an anastomotic leakage in TME by proximal fecal diversion with loop ileostomy has been a subject of debate for many years. It has been suggested that defunctioning ileostomy ameliorates the septic effects of a leak, which potentially leads to pelvic abscess formation and peritonitis [11, 12]. Several randomized trials and comparative studies proposed the creation of protective ileostomy as a means to reduce the risk of AL. However, authors are not unanimous on whether the protective stoma is required in all cases of TME. Unfortunately, despite many risk factors being identified, it is not possible to predict which patients will develop anastomotic leakage [13]. The fundamental question is which patients will benefit from diversion. In fact, in real clinical scenario most patients undergoing TME do not develop leakage at all. For this reason, the question arises whether strategy of elective defunctioning ileostomy in all patients is not an overtreatment in majority of them. In addition, the presence of stoma may also increase the risk of complications related to stoma itself, but also to the subsequent stoma closure [14–16]. On the other hand, anastomotic leakage is not only associated with a prolonged length of hospital stay or increased morbidity, but may also delay postoperative chemotherapy compromising long-term survival [17, 18]. In a review by Hanna et al. authors concluded that creation of defunctioning ileostomy (being the fecal diversion procedure of choice) should be a joint one between the patient and surgeon [19]. However, results of most recently published studies may provide fresh perspective.

Therefore, our aim was to review the current literature to assess the benefits of defunctioning ileostomy creation in patients undergoing anterior rectal resection with TME.

## RESULTS

**Figure 1:**
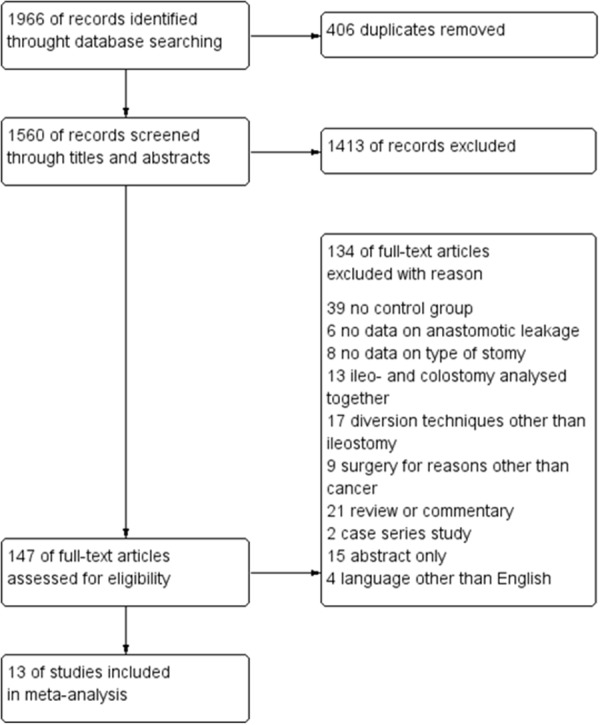
PRISMA flowchart

**Figure 2:**
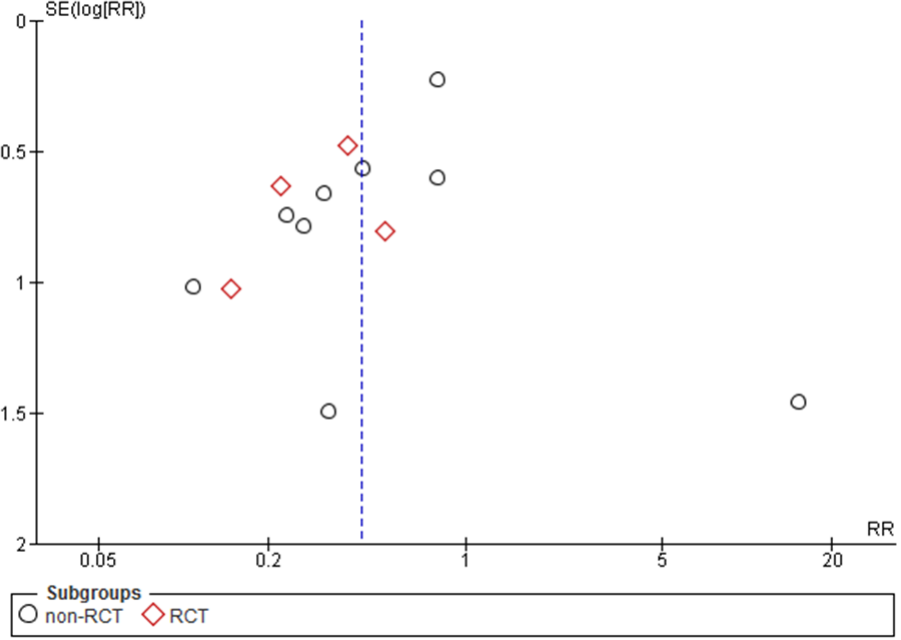
Funnel plot

The initial reference search yielded 1,966 articles. After removing 406 duplicates, 1,560 articles were evaluated through titles and abstracts. This produced 147 papers suitable for full-text review and ultimately, we enrolled 13 studies [20–32]. Out of those, 4 were randomized controlled trials (RCTs) and 9 were comparative studies with a total of 2,366 patients (1,026 with and 1,340 without protective ileostomy). Table 1 presents characteristics of included studies. A PRISMA flowchart of the analyzed studies is presented in Figure 1. The funnel plot of publication bias is presented in Figure 2. The funnel plot is asymmetric due to the increased heterogeneity of included articles. Missing studies in the middle and right of the plot may result from publication bias. However, due to the small number of included articles test power could be too low to distinguish chance from real asymmetry. Quality assessment of studies is presented in Table 1. Additional, more detailed information is included in Supplementary Table 1.

**Table 1:** Baseline characteristics

Study	Year	Type of study	No. of patients in study/ control group	JADAD/NOS quality score	Neoadjuvant therapy in study/ control group (%)	Type of operation laparoscopic/open	Permanent/ End stomy	Type of resection	Definition of anastomotic leakage
**Anderin** **[****20****]**	2015	CS	139/148	7	94.9/76.4	OP	ND	TME	clinical
**Chude** **[****21****]**	2008	RCT	136/120	0	ND	ND	ND	LAR	clinical
**Gong** **[****22****]**	2013	CS	26/36	6	ND	OP	0/26	TME	ND
**Gumbau** **[****23****]**	2015	CS	58/46	5	77.6/36.9	LAP/OP	ND	TME	clinical
**Ihnat** **[****24****]**	2016	CS	78/73	8	35.9/30.1	LAP	4/78	TME	radiological /clinical
**Karahasanoglu** **[****25****]**	2011	CS	23/54	6	21.7/1.8	LAP	3/23	TME/PME	ND
**Kim** **[****26****]**	2015	CS	67/35	6	0/0	LAP/OP	4/67	LAR	clinical
**Maroney** **[****27****]**	2016	CS	57/42	8	75.4/23.8	LAP/OP	5/57	LAR	clinical
**Mrak** **[****28****]**	2016	RCT	94/72	3	61.7/38.9	OP	2/94	TME	clinical
**Seo** **[****29****]**	2013	CS	246/590	7	63.8/24.1	OP	9/246	TME	clinical
**Skrovina** **[****30****]**	2011	CS	50/64	7	ND	LAP	1/50	TME	clinical
**Thoker** **[****31****]**	2014	RCT	34/44	3	ND	OP	3/34	LAR	radiological
**Urlich** **[****32****]**	2009	RCT	18/16	3	83.3/50	OP	ND	TME	radiological /clinical

ND - no data, OP - open approach, LAP - laparoscopic approach, RCT - randomized controlled trial, CS - comparative study, TME – total mesorectal excision, PME – partial mesorectal excision, LAR – low anterior resection.

**Figure 3:**
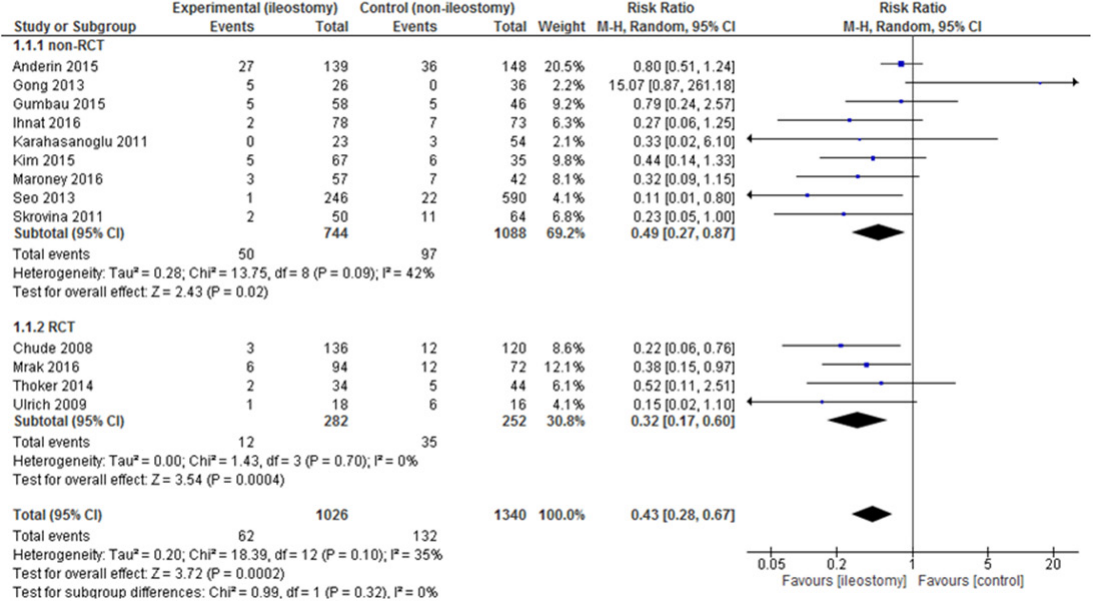
Pooled estimates of anastomotic leakage after rectal resection with versus without defunctioning ileostomy

Anastomotic leakage was reported in all 13 studies. The analysis (Figure 3) showed significant differences among the studied groups, 62/1,026 (6.04%) in the group with a defunctioning stoma vs. 132/1,340 (9.85%) in the group without it: RR=0.43, 95% CI 0.28 - 0.67, p for effect = 0.0002, p for heterogeneity = 0.10, I^2^= 35%. However, when 95% prediction interval was calculated it was not statistically significant [0.14 ; 1.28].

**Figure 4:**
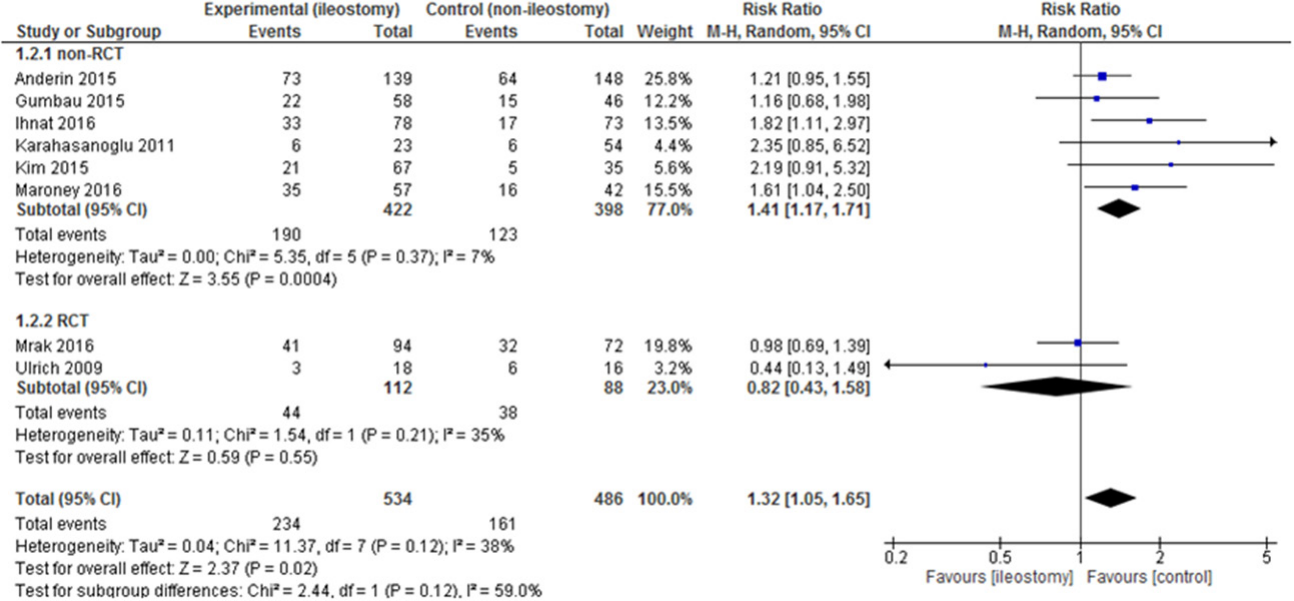
Pooled estimates of complication rate after rectal resection with versus without defunctioning ileostomy

Data on the complication rate were present in 8 included articles. The analysis established significant differences in the complication rate between the group with a defunctioning stoma 234/534 (43.82%) vs. 161/486 (33.13%) in the group without it: RR = 1.32, 95% CI 1.05–1.65, *p* for effect = 0.02, *p* for heterogeneity = 0.12, I^2^ = 38% (Figure 4). In this outcome, 95% prediction interval was not significant [0.77; 2.25].

**Figure 5:**
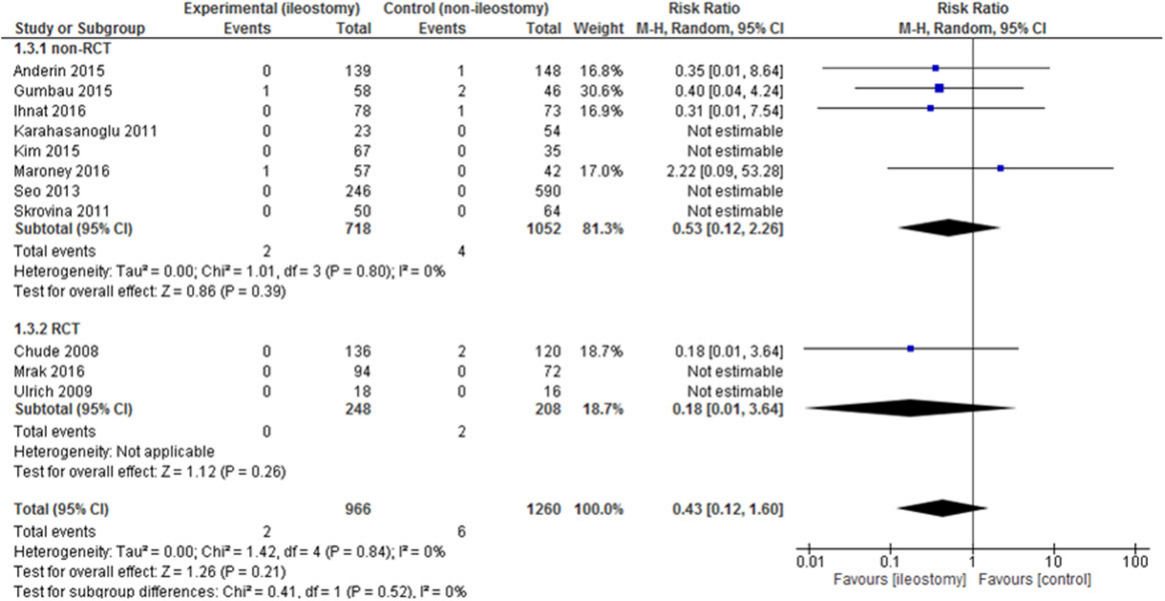
Pooled estimates of mortality after rectal resection with versus without defunctioning ileostomy

Mortality was presented in 11 out of 13 included studies. In 4 papers, 30-day mortality was reported. Chude et al. did not specify for what period of time the mortality was calculated. As many as 6 authors reported no deaths in the analyzed groups. There were no statistically significant differences among the studied groups 2/966 (0.21%) vs. 6/1,260 (0.48%): RR = 0.43, 95% CI 0.12 – 1.60 p for effect = 0.21, p for heterogeneity =0.84, I^2^ =0% (Figure 5).

**Figure 6:**
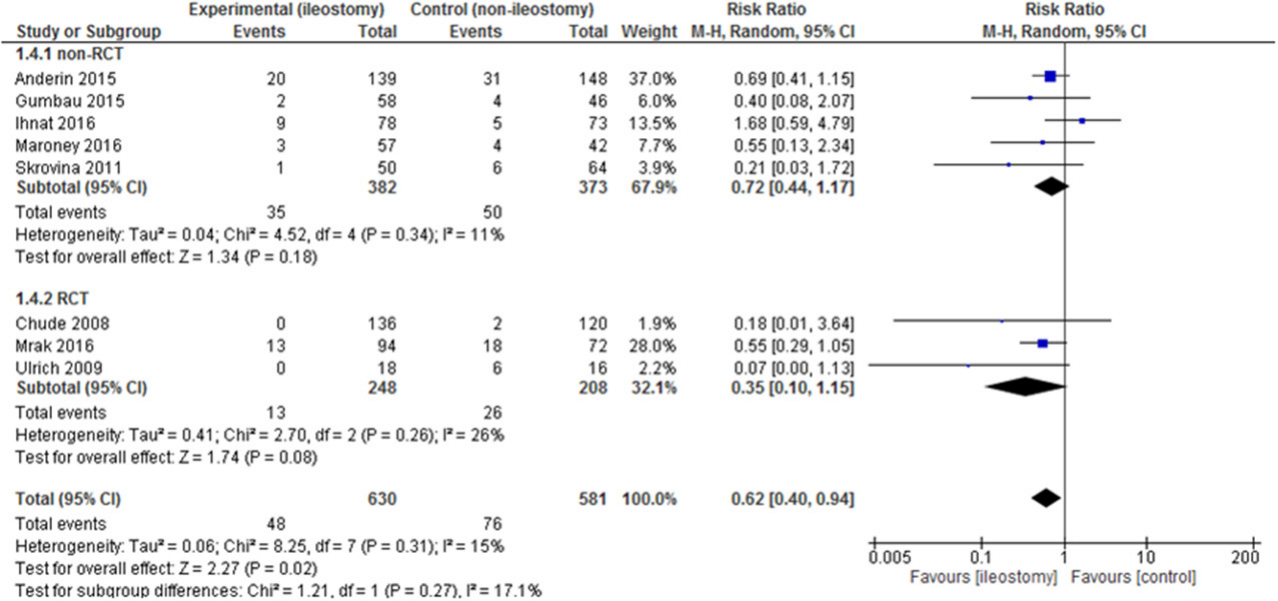
Pooled estimates of reoperations after rectal resection with versus without defunctioning ileostomy

Data on reoperations were present in 8 included articles. The analysis established significant differences in the number of reoperations between patients with vs. without the stoma 48/630 (7.62%) vs. 76/581 (13.08%): RR = 0.62, 95% CI 0.40-0.94, *p* for effect =0.02, *p* for heterogeneity = 0.31, I^2^ =15% (Figure 6). 95% prediction interval for this outcome was 0.18 to 0.97.

**Figure 7:**
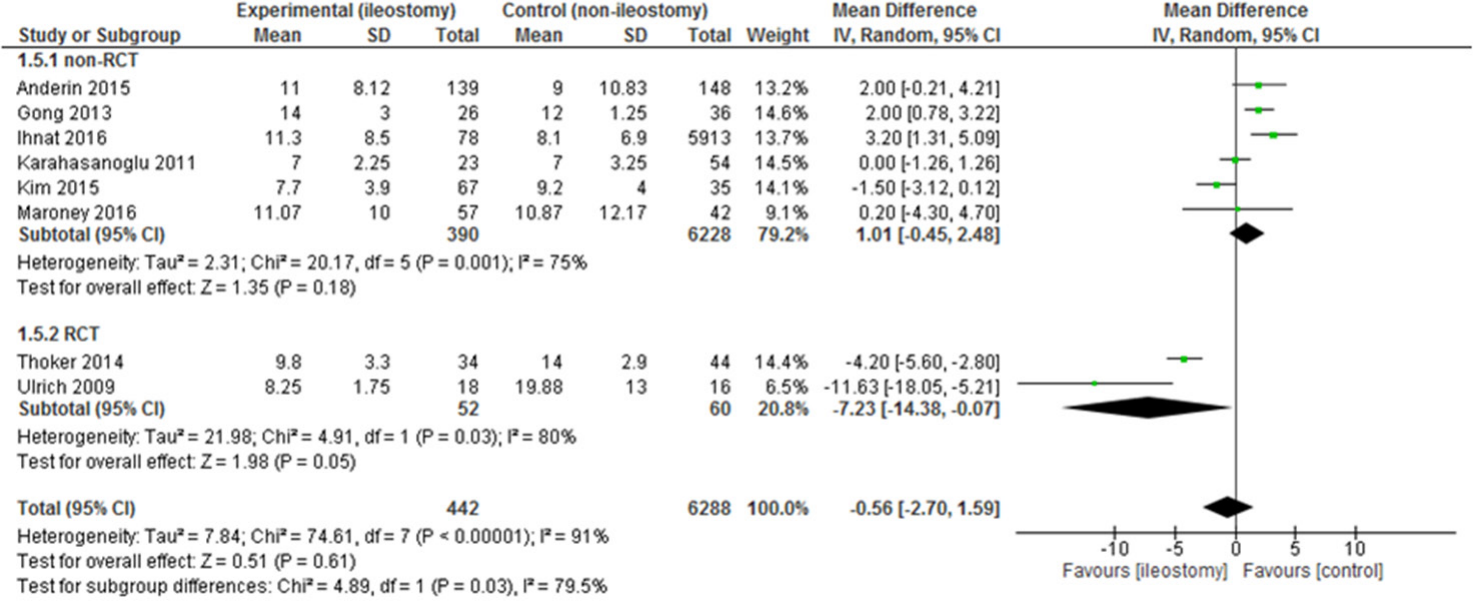
Pooled estimates of length of hospital stay comparing rectal resection with versus without defunctioning ileostomy

The mean length of hospital stay (LOS) was reported in 12 papers. All of them included the primary LOS (excluding potential readmissions). The mean LOS for the group with a defunctioning stoma was 10.1±5.05 days, while for the group without it 10.66±5.96 days. There were no statistically significant variations among the studied groups: mean difference = -0.56, 95% CI -2.70 -1.59, *p* for effect = 0.61, *p* for heterogeneity <0.00001, I^2^ = 91% (Figure 7).

**Figure 8:**
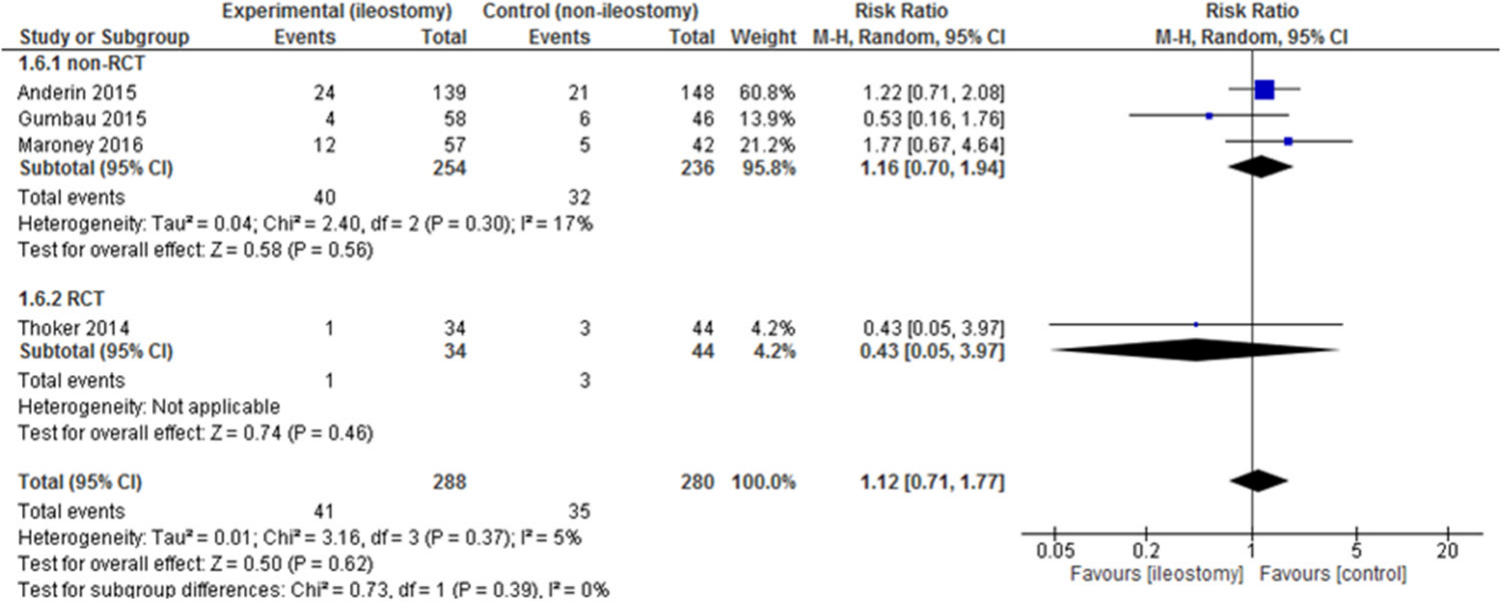
Pooled estimates of readmission rate after rectal resection with versus without defunctioning ileostomy

Readmissions were presented in 4 out of 13 included studies. There were no statistically significant variations among the studied groups 41/288 (14.24%) vs. 35/280 (12.5%): RR = 1.12, 95% CI 0.71 – 1.77, *p* for effect = 0.62, *p* for heterogeneity = 0.37, I^2^ = 5% (Figure 8).

## DISCUSSION

This systematic review, with a meta-analysis, is based on 13 studies (4 RCTs and 9 comparative studies) with more than 2,000 patients. It showed that a defunctioning ileostomy is associated with a decrease in the anastomotic leakage rate and reoperations, an increase of the overall complication rate with no influence on mortality, length of hospital stay and readmission rate.

Nowadays, the role of ileostomy in every anterior rectal resection with TME is still controversial. There are no established guidelines that would impose its creation. Numerous studies are available in the literature, both showing its benefits and its adverse effects. On the one hand, the creation of a protective stoma during primary surgery is intended to lower the rate of clinical anastomotic leakage, which is one of the most severe complications occurring after anterior rectal resection with TME [33, 34]. It can be found in about 10% of patients operated on due to oncological causes and may lead to increased mortality, delays in the introduction of adjuvant therapy, thus disrupt patient treatment leading to worse results [35–37]. On the other hand, a defunctioning ileostomy is associated with more complications (high stoma flow, prolapse, kidney failure, skin excoriation etc.) and some authors report that it also prolongs hospital stay [38, 39]. Additionally, these patients require another surgery, which also involves the risk of complications, and not all patients undergo surgical closure of the stoma [8, 40]. The risk of a defunctioning ileostomy is repeatedly reported by many authors to be in a range from 8% to 25% [41]. Despite the suggestion that ileostomy should be closed within 10–12 weeks, this is not commonly practiced, and the median time to reversal is 30 weeks [42]. Postoperative chemotherapy also prolonged the period of time to reversal and in this group of patients the period of time to stoma closure is up to 40 weeks [43]. Creating a stoma is also associated with higher costs of hospitalization. Floodeen et al. showed that a defunctioning stoma in low anterior resection was associated with higher costs for 5 years after surgery, despite the cost-savings associated with a reduced frequency of anastomotic leakage [44]. These results seem to be significant, especially since more than 90% of patients do not benefit from the stoma [45]. Another solution could be creating a ghost ileostomy. In this group of patients it is possible to create a loop ileostomy only in case of anastomotic leakage and without the need for laparotomy. However, even pioneers of this technique recommend it only in patients with low and medium risk of this complication [46, 47]. We have observed that creating a protective stoma may be beneficial in reducing both the rate of anastomotic leakage and reoperations. In order to evaluate significant results of random-effects data pooling we have used prediction intervals to fully estimate their clinical application. The prediction interval for anastomotic leakage was 0.14-1.28, thus the clinical application may be restricted in some settings. However, it has been shown clinically relevant in terms of reoperations. Leakage after rectal resection with TME is one of the most serious complications. It is due to the fact, that the large proportion of these patients require reoperation. In addition it is associated with relatively high mortality risk [48, 49]. Many patients with leakage in the group without a stoma require conversion to colostomy (Hartmann procedure) that in some cases is permanent. The percentage of patients with a permanent stoma in the included studies is presented in Table 1 and ranges between 0% and 13% [22, 25]. What is worth mentioning is that ileostomy allows in many patients an effective treatment of leaks, including endoVAC therapy which is highly successful in this indication [50–52].

One of the well-known risk factors for leaks is neoadjuvant treatment [53, 54]. In the analyzed studies, patients with neoadjuvant treatment were more often treated with anastomosis accompanied by a protective stoma than not (67.7% vs. 32.8%), which may create a bias. Even though the higher proportion of patients with a stoma had neoadjuvant treatment, the proportion of leaks in this group was smaller than in the group without a protective stoma. This speaks for benefits of fecal diversion.

The overall complication rate was higher in the group of patients with a defunctioning ileostomy compared to patients without it. The prediction interval for morbidity ranged from 0.77 to 2.25 meaning that not in every clinical situation patients with stoma may be at greater risk of developing complications. Despite the decreased leakage rate in the protective stoma group, there is a whole range of stoma-related complications mentioned previously. On the one hand, a protective stoma reduces the incidence of leakage (one of the most severe complications). On the other hand, it leads to a higher incidence of complications (mainly less severe) [55–57]. It seems, however, that the patient with a stoma still benefits. Presence of stoma reduces one of the heaviest complications – leakage which mostly requires reoperation. The higher morbidity rate results mainly from an increased number of less severe complications, requiring mostly only pharmacological treatment and a slightly longer hospital stay. Mostly, they do not affect the delay of adjuvant treatment. We have not been able to provide a more detailed division of complications with a separate analysis due to lack of detailed data.

In our meta-analysis there were no significant differences in the mortality rate between the groups. The mortality rate after anterior rectal resection with TME reported in the literature ranges between 1% to 8% and rises to 6-22% when anastomotic leakage occurs [8, 58]. In our analysis it was much lower – nearly 0.2% in the stoma group and 0.5% in the group without it. Another interesting consideration is whether stoma creation affects long-term outcomes, including the 5-year survival. This assessment is, however, very difficult, mainly due to a large number of disturbing factors. The length of hospital stay and readmission rate did not differ significantly between the analyzed groups.

One of the limitations of this study is the variability of the quality of the included studies. We included both randomized and non-randomized studies. Besides, we were not able to fully evaluate types of rectal resections performed. In 8 studies the authors reported them as TME, whereas in 4 it was low anterior resection (LAR) and in one study both TME and partial total mesorectal excision (PME) in cases of cancer of the upper rectum. However, taking into consideration the relatively recent date of publication we may assume that all cases of LAR were performed according to TME standards at the same time. The majority of trials were clinical control studies, only 4 were RCTs. Comparative studies have a potential risk of selection bias – a surgeon could decide to create a defunctioning stoma in the case of patients with a higher risk for AL. Another cause of bias may be the difference in the height of the anastomosis, that is the length between the anal verge and anastomosis. It is not reported by all authors. It seems, however, that lower anastomosis is associated with a greater risk of anastomotic leakage [9, 11]. The included studies differed in the percentage of neoadjuvant treatment as mentioned above. In addition, the large heterogeneity of the included studies restrains us from drawing strong conclusions in some results. Despite numerous studies on the subject, it appears that further well-designed randomized studies separately with or without neoadjuvant treatment are needed to better define the group of patients that would need a defunctioning ileostomy and perhaps establishing a standard for its selective use. Additionally the results should be considered with caution since evaluation of funnel plot revealed publication bias.

This meta-analysis shows on the large population that a defunctioning ileostomy may decrease the anastomotic leakage rate, additionally significantly reducing the risk of reoperation but it may also increase the overall complication rate. Creating or no protective stoma has no effect on mortality, the length of hospital stay and readmission rate.

## MATERIALS AND METHODS

### Study selection

A search was conducted by two researchers (NG, MW) in October 2017, using the Medline (through Ovid and PubMed), Embase and Cochrane databases to identify all eligible studies with language restricted to English. The search terms used were “ileostomy”, “stomy”, “cancer”, “adenocarcinoma”, “tumor”, “malignancy”, “neoplasm”, “defunctioning”, “diverting”, “protective”, “loop”, “rectal” and their abbreviations with the addition of Boolean operators “AND” and “OR”. References of all retrieved articles were checked for potentially eligible articles.

### Data extraction

Four researchers (NG, MW, MM, JW) identified and selected citations from the search independently. Each citation was assessed by at least two researchers. In the event of uncertainties relating to inclusion, a third reviewer was consulted (MP) until consensus was reached. Extraction of data from every selected article was conducted by at least two researchers. Basic information regarding the included studies were first author, year of publication, study design, number of patients in each group and type of operation (laparoscopic/open).

### Inclusion and exclusion criteria

The inclusion criteria were as follows: (1) studies comparing patients with vs. without a defunctioning ileostomy in rectal resection (2) data on anastomotic leakage in both groups, (3) paper in English.

Studies were excluded when: (1) full extraction was not possible (2) review or meta-analysis (3) decompression techniques other than ileostomy, (4) single group studies.

In the case of studies comprising the same patient cohort, only the most recent or complete study was included (Table 1).

### Outcomes of interest

The primary outcome of interest was the anastomotic leakage rate. Secondary outcomes involved short-term outcomes: complication rate, mortality, reoperation rate, length of hospital stay and 30-day readmission.

### Statistical analysis

Analysis was performed using RevMan 5.3 (freeware from the Cochrane Collaboration). Statistical heterogeneity and inconsistency were measured using Cochran’s Q tests and I2, respectively. Qualitative outcomes from individual studies were analyzed to assess individual and pooled risk ratios (RR) with pertinent 95% confidence intervals (CI) favoring surgery with a protective ileostomy over surgery without it. When the study included medians and interquartile ranges, we calculated the mean ± SD using a method proposed by Hozo et al. [59]. Weighted mean differences (WMD) with 95% CI were presented for quantitative variables using the inverse variance random-effects method. Statistical significance was observed with two-tailed 0.05 level for hypothesis and with 0.10 for heterogeneity testing, while unadjusted p-values were reported accordingly. Clinical application of significant results acquired with random-effects data pooling was evaluated using prediction intervals according to Riley et al. [60].

Non-randomized studies were evaluated with the Newcastle–Ottawa Scale (NOS), which consists of three factors: patient selection, comparability of the study groups and assessment of outcomes. A score of 0 to 9 was assigned to each study and studies achieving a score of 6 or greater were considered high quality. Randomized studies were assessed with the Jadad scale. This study was performed according to the Preferred Reporting Items for Systematic reviews (PRISMA) guidelines and MOOSE consensus statement [61, 62].

## SUPPLEMENTARY MATERIALS TABLE


