# Supplementary material for: Defunctioning ileostomy reduces leakage rate in rectal cancer surgery - systematic review and meta-analysis
Source: Oncotarget. 2018 Apr 17;9(29):20816–25. doi: 10.18632/oncotarget.25015 (PMC5945534; doi:10.18632/oncotarget.25015)
Supplement: Supplementary file 1 [file oncotarget-09-20816-s001.pdf]

# Defunctioning ileostomy reduces leakage rate in rectal cancer surgery - systematic review and meta-analysis

## SUPPLEMENTARY MATERIALS

**Supplementary Table 1: Assessment of the quality of included studies according to Jadad scale (randomized trials) or Newcastle-Ottawa Scale (non-randomized studies)**

| Study | Year | Study design | Jadad scale | | | | | | Newcastle-Ottawa Scale | | | |
|---|---|---|---|---|---|---|---|---|---|---|---|---|
| | | | **Jadad Scale (total)** | **Was the study described as random?** | **Was the randomization scheme described and appropriate?** | **Was the study described as double-blind?** | **Was the method of double blinding appropriate?** | **Was there a description of dropouts and with drawals?** | **NOS (total)** | **NOS Selection** | **NOS Comparability** | **NOS Out come** |
| Anderin [20] | 2015 | CS | | | | | | | **7** | 3 | 1 | 3 |
| Chude [21] | 2008 | RCT | **0** | 1 | -1 | 0 | 0 | 0 | | | 1 | 3 |
| Gong [22] | 2013 | CS | | | | | | | **6** | 2 | 1 | 2 |
| Gumbau [23] | 2015 | CS | | | | | | | **5** | 2 | 2 | 3 |
| Ihnat [24] | 2016 | CS | | | | | | | **8** | 3 | 2 | 1 |
| Karahasanoglu [25] | 2011 | CS | | | | | | | **6** | 3 | 1 | 3 |
| Kim [26] | 2015 | CS | | | | | | | **6** | 2 | 2 | 3 |
| Maroney [27] | 2016 | CS | | | | | | | **8** | 3 | | |
| Mrak [28] | 2016 | RCT | **3** | 1 | 1 | 0 | 0 | 1 | | | 1 | 3 |
| Seo [29] | 2013 | CS | | | | | | | **7** | 3 | 1 | 3 |
| Skrovina [30] | 2011 | CS | | | | | | | **7** | 3 | | |
| Thoker [31] | 2014 | RCT | **3** | 1 | 1 | 0 | 0 | 1 | | | | |
| Urlich [32] | 2009 | RCT | **3** | 1 | 1 | 0 | 0 | 1 | | | | |

Assesment of the quality of included studies according to Jadad scale (randomized trials) or Newcastle-Ottawa Scale (non-randomized studies).
RCT -randomized controlled trial, CS – comparative study, NOS - The Newcastle-Ottawa Scale.
